# Association between urinary polycyclic aromatic hydrocarbons and hypertension in the Korean population: data from the Second Korean National Environmental Health Survey (2012–2014)

**DOI:** 10.1038/s41598-020-74353-w

**Published:** 2020-10-13

**Authors:** Tae-Woo Lee, Dae Hwan Kim, Ji Young Ryu

**Affiliations:** Department of Occupational and Environmental Medicine, Inje University Haeundae Paik Hospital, 875 Haeundae-ro, Haeundae-gu, Busan, 48108 South Korea

**Keywords:** Environmental sciences, Diseases, Medical research, Risk factors

## Abstract

Polycyclic aromatic hydrocarbons (PAHs) are environmental and occupational pollutants derived from incomplete combustion of organic materials, including wood and fossil fuels. Epidemiological studies have evaluated the association between PAH exposure and hypertension or cardiovascular disease in the general population, but the evidence is limited. In this study, we evaluated the association between urinary PAH metabolites and hypertension in the Korean adult population. A total of 6478 adults who participated in the Second Korean National Environmental Health Survey (2012–2014) were included. The differences in urinary concentrations of four PAH metabolites, including 1-hydroxypyrene, 2-hydroxyfluorene, 1-hydroxyphenanthrene, and 2-naphthol, were compared according to hypertension status using a general linear model. Adjusted odds ratios (aORs) for hypertension were calculated according to the quartile groups of urinary PAH metabolites after adjusting for age, sex, body mass index (BMI), smoking, and alcohol consumption in multiple logistic regression analyses. The estimated mean concentrations of urinary 1-hydroxyphenanthrene were significantly higher in the hypertension group than in the non-hypertension group. In 1-hydroxyphenanthrene, the OR for hypertension was significantly higher in the third and fourth quartile groups than in the first quartile group (third: OR 1.707, 95% CI 1.203–2.423, fourth: OR 1.604, 95% CI 1.158–2.223). No significant associations were detected for the other metabolites. Our results suggest an association between exposure to PAHs and hypertension in a Korean adult population. Further studies are required to evaluate the effects of low-dose long-term exposure to PAHs on hypertension and cardiovascular disease.

## Introduction

Polycyclic aromatic hydrocarbons (PAHs) are environmental and occupational pollutants derived from incomplete combustion of organic materials, including wood and fossil fuels. PAHs are emitted from gasoline and diesel engines, thermal power plants, waste incinerators, residential heaters, tobacco smoke, and industries such as coke and carbon production and petroleum refining^1^. Cooking fumes are also a source of PAHs^2^. PAHs are ubiquitous and persistent organic pollutants (POPs)^3^.

Long-term exposure to PAHs can have adverse effects on human health. Epidemiological studies have reported associations between PAH exposure and lung, bladder, and skin cancers^4^. The International Agency for Research on Cancer (IARC) classified benzo(a)pyrenes as group 1 carcinogens (carcinogenic to humans)^5^. In addition to being carcinogenic, PAHs may have other significant implications for health.

A cohort study reported a positive association between occupational exposure to PAHs and ischemic heart disease^6^. In another study, long-term occupational exposure to PAHs was related to the development of atherosclerosis among taxi drivers^7^. Several studies have reported that exposure to PAHs can elevate blood pressure, which is a key risk factor for cardiovascular disease (CVD)^8,9^. PAHs are absorbed onto particulate matter (PM) such as PM10 and PM2.5 in ambient air^10^, and epidemiological studies have documented associations between ambient PM and CVDs, including hypertension^11–15^. Increased oxidative stress, subsequent vasoconstriction, endothelial dysfunction, and altered circadian rhythms have been suggested as mechanisms of the effects of PAHs on blood pressure or CVDs^16^.

Because PAHs are widespread in the environment and disperse in ambient air, exposure can be constant and may cause significant health problems, including CVDs. Few studies have evaluated the association between PAH exposure and hypertension or CVDs in the general population, so the evidence is limited. In this study, we evaluated the association between urinary PAH metabolites and hypertension in a Korean adult population.

## Methods

### Study participants

We used data from the Second Korean National Environmental Health Survey (KoNEHS) conducted by the National Institute of Environmental Research from 2012 to 2014. The survey applied a stratified sampling using the 2010 national population and housing census. The survey participants consisted of 6478 subjects from 400 districts who were chosen to be proportional to the population distribution. The data were collected through person-to-person interviews and biological sampling. Informed consent was obtained from all subjects in KoNEHS and we used publicly available de-identified data only. This study was approved by the Institutional Review Board of Haeundae Paik Hospital (IRB No. 2020-03-013). All research reported in this manuscript was performed in accordance with relevant guidelines and regulations.

### Hypertension and covariates

A medical questionnaire was administered during the personal interview to ascertain participants’ medical history, including any history of hypertension. Participants were asked “Do you have cardiovascular disease?” If they answered “yes” to the question and reported hypertension as a detailed diagnosis, they were considered hypertensive in the current study.

Age, sex, body mass index (BMI), smoking status, and drinking status were included as variables. Smoking status was classified into three groups: current smoker, ex-smoker, and nonsmoker. Drinking status was classified into two groups: drinking and non-drinking.

### PAH exposure: urinary concentrations of PAH metabolites

Exposure to PAHs was evaluated with concentrations of four urinary PAH metabolites: 2-naphthol, 1-hydroxyphenanthrene, 2-hydroxyfluorene, and 1-hydroxypyrene. Urinary concentrations of these PAH metabolites were divided by the concentration of urine creatinine to correct for variability due to differences in urinary output (µg/g creatinine). Urinary creatinine was analyzed by a colorimetric method using a Creatinine (SIEMENS/USA) reagent and ADVIA 1800 (SIEMENS/USA) equipment, and the concentration unit is g/L. Each urinary PAH metabolite was grouped by quartile: quartile 1 (≤ 25th percentile) vs. quartile 2 (> 25th and ≤ 50th percentile) vs. quartile 3 (> 50th and ≤ 75th percentile) vs. quartile 4 (> 75th percentile).

Urinary PAH metabolites were analyzed by the following methods in KoNEHS^17–19^. Random spot urine samples were collected once from all 6478 subjects. All subjects were guided to collect more than 65 cc of midstream urine. Spot urine specimens were transferred to the laboratory in an ice box and stored at − 20 °C prior to analysis. All urinary PAH metabolite concentrations were analyzed by gas chromatography-mass spectrometry (GC–MS). The metabolites were hydrolyzed with β-glucuronidase/arylsulfatase followed by derivatization with bistrimethylsilyl trifluoroacetamide. Concentrations were calculated from calibration curves drawn by the standard addition method. The limits of detection (LOD) were 0.015 µg/L for 1-hydroxypyrene, 0.05 µg/L for 2-naphthol, 0.047 µg/L for 1-hydroxyphenanthrene, and 0.04 µg/L for 2-hydroxyfluorene. The target coefficient (R^2^) of the calibration curve was ≥ 0.995 for internal quality control. Measurements less than the LOD were replaced with the LOD divided by the square root of 2.

### Statistical analyses

In our analyses, strata, clusters, and sampling weights were applied to account for the stratified two-stage cluster sampling design of the KoNEHS. Estimated means and percentages were calculated for demographic characteristics. Because the distributions of the urinary concentrations of PAH metabolites were skewed, all metabolites were log-transformed before analyses. The general linear model (GLM) was used to analyze the difference in urinary PAH metabolite concentrations by hypertension status. Adjusted odds ratios (aORs) for hypertension by PAH exposure group were calculated with multiple logistic regression analyses. The effect of PAH exposure mixture on hypertension was estimated by quantile g-computation model^20^. Additional sensitivity analyses were conducted to explore associations of urinary PAHs metabolisms with hypertension by smoking status and gender. SPSS (version 25 for Windows; IBM, Armonk, NY, USA) was used for statistical analyses. Quantile g-computation regression was conducted using the qgcomp package (version 2.5.0) with R studio (R version 4.0.2; R Foundation for Statistical Computing, Vienna, Austria.). *p* < 0.05 was considered significant.

## Results

The demographic characteristics of the study population are shown in Table 1. The estimated mean with standard error or the sample size (%) of each demographic variable is listed. The total number of participants was 6478. The response rate for the questionnaire item related to CVDs was 46.2% (n = 2999). Of respondents who answered, 41.1% said that they had hypertension (n = 1016).

**Table 1:** Demographic characteristics of the study population (n = 6478).

Variable	Estimated mean ± SE or sample size (%)
Age (years)	46.3 ± 0.4
**Sex**	
Male	3187 (49.2)
Female	3291 (50.8)
BMI (kg/m^2^)	24.1 ± 0.1
**Smoking**	
Nonsmoker	4064 (62.7)
Ex-smoker	1022 (15.8)
Current smoker	1392 (21.5)
**Drinking**	
Yes	2285 (35.3)
No	4193 (64.7)
Hypertension^a^	1016 (41.1)

^a^The response rate for the questionnaire item related to cardiovascular disease was 46.2% (n = 2999).

The estimated percentiles of the urinary metabolites and PAHs (ng/g creatinine) are listed in Table 2. We analyzed concentrations of four urinary PAH metabolites (2-naphthol, 1-hydroxyphenanthrene, 2-hydroxyfluorene, and 1-hydroxypyrene) and estimated the 5th, 25th, 50th, 75th, and 95th percentiles of each.

**Table 2:** Estimated percentiles of the urinary polycyclic aromatic hydrocarbon metabolites (ng/g creatinine).

Metabolite	5th percentile	25th percentile	50th percentile	75th percentile	95th percentile
1-Hydroxypyrene	60	128	201	312	652
2-Hydroxyfluorene	92	188	310	705	2052
1-Hydroxyphenanthrene	40	78	121	193	381
2-Naphthol	543	1328	2802	7394	20,636

Table 3 shows the estimated geometric mean urinary PAH metabolite concentrations according to the presence or absence of hypertension. Each mean is listed with the 95% confidence interval. GLM analyses revealed that the mean for 1-hydroxyphenanthrene was significantly higher in the hypertensive group than in the non-hypertensive group (*p* < 0.001).

**Table 3:** Estimated geometric means for urinary polycyclic aromatic hydrocarbon metabolites (µg/g creatinine) by hypertension.

Metabolite	Mean (95% CI)		*p*-value
	HTN(−)	HTN( +)	
1-Hydroxypyrene	0.216	0.222	0.531
95% CI	0.203–0.230	0.208–0.236	
2-Hydroxyfluorene	0.356	0.385	0.124
95% CI	0.331–0.382	0.353–0.419	
1-Hydroxyphenanthrene	0.128	0.152	< 0.001
95% CI	0.121–0.135	0.144–0.160	
2-Naphthol	3.235	3.219	0.930
95% CI	2.975–3.518	2.942–3.522	

*HTN* hypertension.

Table 4 lists the results of multiple logistic regression analyses of urinary PAH metabolites and hypertension. We show the adjusted ORs of the second, third, and fourth quartile groups of each metabolite for hypertension compared to the reference group (first quartile group). The ORs of the third and fourth quartile groups of 1-hydroxyphenanthrene were significantly higher than that of the first quartile group (third: OR 1.707, 95% CI 1.203–2.423; fourth: OR 1.604, 95% CI 1.158–2.223; *p* for trend 0.002). Also, there was a significant relationship between continuous level of 1-hydroxyphenanthrene and hypertension (OR 1.241, 95% CI 1.063–1.449). No significant associations were observed for the other metabolites.

**Table 4:** Multiple logistic regression analyses of urinary polycyclic aromatic hydrocarbon metabolites and hypertension.

Metabolite	Adjusted odds ratio^a^	95% confidence interval	*p* for trend
**1-Hydroxypyrene**			0.716
≤ 25th percentile	1 (Reference)		
25–50th percentile	1.072	0.742–1.548	
50–75th percentile	1.235	0.882–1.728	
> 75th percentile	0.876	0.626–1.227	
Continuous^b^	0.912	0.791–1.052	
**2-Hydroxyfluorene**			0.568
≤ 25th percentile	1 (Reference)		
25–50th percentile	1.152	0.807–1.645	
50–75th percentile	0.934	0.667–1.308	
> 75th percentile	1.292	0.839–1.989	
Continuous^b^	1.105	0.949–1.287	
**1-Hydroxyphenanthrene**			0.002
≤ 25th percentile	1 (Reference)		
25–50th percentile	1.334	0.930–1.916	
50–75th percentile	1.707	1.203–2.423	
> 75th percentile	1.604	1.158–2.223	
Continuous^b^	1.241	1.063–1.449	
**2-Naphthol**			0.634
≤ 25th percentile	1 (Reference)		
25–50th percentile	0.944	0.696–1.280	
50–75th percentile	0.876	0.657–1.169	
> 75th percentile	0.967	0.699–1.339	
Continuous^b^	0.963	0.879–1.055	

^a^Adjusted for age, sex, BMI, smoking status, and drinking status.

^b^Log-transformed concentration of urinary PAH metabolite.

**Figure 1:**
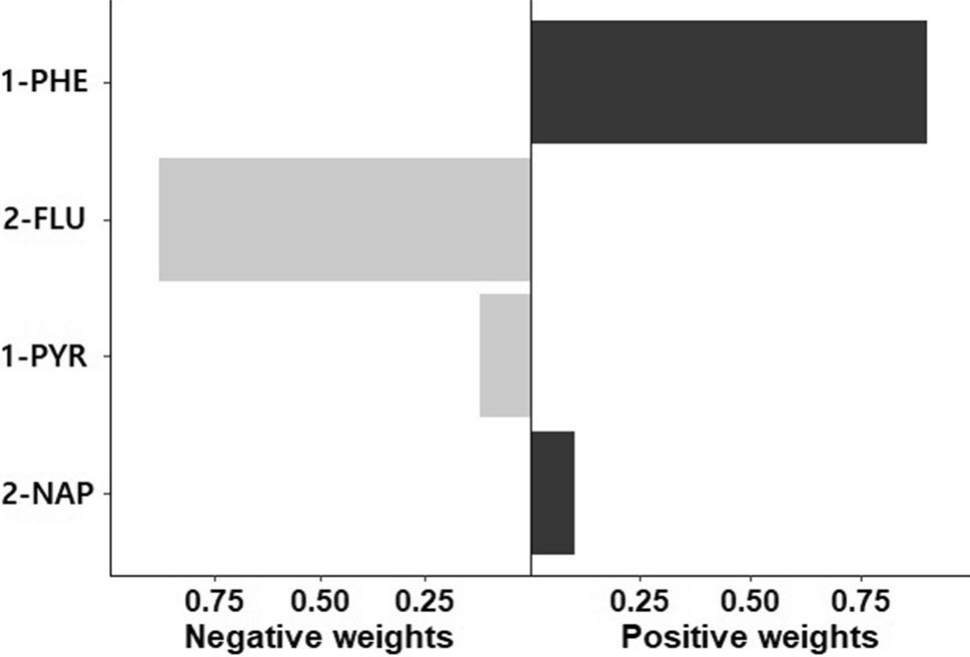
Weights of each PAH metabolite by quantile g-computation regression for hypertension. *1-PHE* 1-Hydroxyphenanthrene, *2-FLU* 2-hydroxyfluorene, *1-PYR* 1-hydroxypyrene, *2-NAP* 2-naphthol.

Table 5 shows the mixture effect of PAH exposure for hypertension. In the quantile g-computation model, there was no statistically significant association between hypertension and the mixture of four PAH metabolites. The weights for each PAH metabolite by the quantile g-computation regression, are shown in Fig. 1. The highest weight for the positive direction was 1-Hydroxyphenanthrene (89.9%).

**Table 5:** Association between hypertension and the mixture of four PAH metabolites.

	Odds ratio	95% confidence interval	*p*-value	Positive^a^	Negative^b^
Hypertension	1.091	0.963–1.235	0.168	0.117	− 0.0297

Quantile g-computation model; adjusted for age, sex, BMI, and smoking status.

^a^Sum of positive coefficients.

^b^Sum of negative coefficients.

Additionally, we analyzed the potential for effect measure modification by sex and smoking for the association between 1-hydroxyphenanthrene and hypertension (Table 6). In men, the ORs of the third and fourth quartile groups of 1-hydroxyphenanthrene were significantly higher than that of the first quartile group (third: OR 2.198, 95% CI 1.325–3.649; fourth: OR 1.991, 95% CI 1.183–3.350). By smoking status, there were dose response relationships between 1-hydroxyphenanthrene and hypertension in nonsmokers and current smokers, with the significant association observed in the third quartile group among ex-smokers (OR 2.282, 95% CI 1.093–4.762).

**Table 6:** Associations between urinary 1-hydroxyphenanthrene and hypertension by gender and smoking status.

Potential covariate	1-Hydroxyphenanthrene	Adjusted odds ratio^a^	95% confidence interval	*p*-value
**Gender**^a^				
Men (n = 3187)	≤ 25th percentile	1 (Reference)		
	25–50th percentile	1.368	0.834–2.243	0.214
	50–75th percentile	2.198	1.325–3.649	0.002
	> 75th percentile	1.991	1.183–3.350	0.010
Women (n = 3291)	≤ 25th percentile	1 (Reference)		
	25–50th percentile	1.219	0.720–2.062	0.460
	50–75th percentile	1.185	0.723–1.945	0.500
	> 75th percentile	1.207	0.780–1.869	0.397
**Smoking status**^b^				
Nonsmoker (n = 4064)	≤ 25th percentile	1 (Reference)		
	25–50th percentile	1.400	0.873–2.245	0.163
	50–75th percentile	1.437	0.905–2.281	0.124
	> 75th percentile	1.509	0.985–2.311	0.058
Ex-smoker (n = 1022)	≤ 25th percentile	1 (Reference)		
	25–50th percentile	1.030	0.547–1.940	0.927
	50–75th percentile	2.282	1.093–4.762	0.028
	> 75th percentile	1.279	0.664–2.465	0.461
Current smoker (n = 1392)	≤ 25th percentile	1 (Reference)		
	25–50th percentile	1.468	0.361–5.965	0.591
	50–75th percentile	2.041	0.566–7.351	0.274
	> 75th percentile	2.145	0.600–7.662	0.239

^a^Adjusted for age, BMI, smoking status, and drinking status.

^b^Adjusted for age, sex, BMI, and drinking status.

## Discussion

We examined the associations between PAHs and hypertension in the Korean adult population. Among urinary PAH metabolites, 1-hydroxyphenanthrene was positively associated with hypertension independent of smoking status and other confounders, whereas no associations were observed for 2-naphthol, 2-hydroxyfluorene, or 1-hydroxypyrene. The mixture of four PAH metabolites did not show statistically significant relationship with hypertension.

Previous studies have explored the association between PAH exposure and hypertension in humans. A positive dose–response relationship was observed for urinary 2-naphthol and 2-hydroxyphenanthrene and hypertension in the U.S. population^21^. Another U.S. population-based study reported that urinary 4-hydroxyphenanthrene is associated with hypertension^8^. In a study that used data from the Mexican American Cohort Study, a dose-dependent association was detected between ambient levels of PAHs and hypertension among the enrolled Mexican-originating subjects in 2001–2003^9^. Similarly, we observed that 1-hydroxyphenanthrene was positively associated with hypertension in the Korean population.

PAHs are metabolized by cytochrome P450 and aldo–keto reductases into PAH *o*-quinones. The pathway of PAH activation produces reactive oxygen species (ROS) and amplifies ROS through redox cycling^22^. Oxidative stress related to PAH exposure may be associated with hypertension. Nitric oxide (NO) produced by endothelial cells is involved in the regulation of vascular tone and blood pressure^23^. Oxygen free radicals inactivate endothelial-derived NO, which induces vascular relaxation and may impair the vasomotor function of the vascular endothelium^24^. PAHs can also affect blood pressure via the aryl hydrocarbon receptor (AhR)-dependent pathway. Some PAHs bind to the AhR with high affinity^25^ and activate AhR-dependent pathways, causing an increase in intracellular Ca^2+^, which is an important second messenger that regulates blood pressure^26^. An ex vivo study revealed that overexpressing the AhR impairs the activation of endothelial nitric oxide synthase and suppresses NO production^27^.

In addition to hypertension, the association between PAH exposure and CVDs has been studied. Many epidemiological studies have found significant associations between PAH exposure and CVDs, as well as major risk factors predisposing for CVDs, including elevated blood pressure^28^. Preclinical studies have suggested that PAHs such as benzo[a]pyrene, benzo[e]pyrene might have an atherogenic effect. Their atherogenic effect can act by causing an inflammatory process related to an influx of inflammatory cells such as T lymphocytes into plaques^29^. Population-based studies also support the atherogenic effects of PAHs by revealing an association between PAHs and inflammation^30^.

Among PAHs, pyrene and fluorene are a major component of particulate matter (PM) and PM-related studies have also explored associations with hypertension or CVDs. Previous researches have found that an increase of 10 µg/m^3^ in PM2.5 (particulate matter 2.5 microns or less in aerodynamic diameter) can elevate nearly 1–5 mmHg of blood pressure^12^ and also were associated with 76% increase in CVD risk and 3–76% increase in CVD related mortality^13^. PM2.5 exposure is associated with endothelial dysfunction or systemic inflammation in groups at risk for CVD such as diabetes and ischemic heart disease patients^31,32^. Recent studies showed that environmental exposure to PM2.5 can be related to endothelial injury and systemic inflammation even in young healthy adults^33^. Another study revealed that diesel exhaust increases systolic blood pressure in healthy participants^34^. Furthermore, many studies have shown that air pollution can cause hypertension^35^, and long-term exposure to PM2.5 is related with reduced NO-mediated endothelial function which is a major mechanism of hypertension^36^.

Coke oven workers and chimney sweeps are exposed to high levels of PAHs. Coke oven emissions are associated with hypertension and abnormal electrocardiographic parameters^37^. One study showed that PAH metabolites including 2-hydroxyphenanthrene, 3-hydroxybenzo[a]pyrene, and 3-hydroxy-benzo[a]anthracene are associated with increased diastolic blood pressure in chimney sweeps^38^. These results related to occupational exposure indicate that PAH exposure is associated with increased risk for hypertension.

Our study did not show the association between the mixture of four PAH metabolites and hypertension, and only 1-hydroxyphenanthrene showed a significant association with hypertension among four metabolites. Each PAH analyzed in this study may have different effects on humans, either directly or indirectly, which should be remembered when considering why only 1-hydroxyphenanthrene produced a significant result. For example, in vitro studies suggest that phenanthrene and naphthalene can activate peroxisome/proliferator-activated receptors (PPARs)^39^. In addition, regarding hormonal effects, hydroxylated naphthalene metabolites have potential estrogenic effects, phenanthrene and fluoranthrene have anti-androgenic effects, and 1- and 2-hydroxynaphthalene can act as thyroid hormone antagonists^40–42^.

This is the first study to assess the relationship between exposure to PAHs and hypertension in a Korean adult population and increases our understanding on the effects of PAHs on hypertension. However, our work has certain limitations. First, we lacked data on the long-term effects of PAHs on hypertension, because the KoNEHS was a cross-sectional observational study. Therefore, it was difficult to evaluate the causal relationship between exposure to PAHs and hypertension. Second, the response regarding CVDs was unclear in the KoNEHS questionnaire. Therefore, we could not evaluate the relationship between PAHs and CVDs. Third, other metabolites of PAHs, such as 2-hydroxyphenanthrene and 9-hydroxyfluorene, were not measured in the KoNEHS. Thus, this study was limited to evaluating associations with the four metabolites studied here.

The health risks posed by air pollutants, including PM and PAHs, constitute a public health concern. We evaluated the effects of PAHs on hypertension in the Korean population. Future studies should address the limitations of our present work.

## Conclusion

Our results suggest an association between exposure to PAHs and hypertension in a Korean adult population. Further studies are required to evaluate the effects of low-dose long-term exposure to PAHs on hypertension and CVDs.

## Data Availability

The datasets analyzed during the current study are available on request at the National Institute of Environmental Research, Environmental Health Research Department, https://www.nier.go.kr/NIER/kor/op/nier-op-16.do?menuNo=11000.
